# Three-dimensional printed customized uncemented unipolar prosthesis combined with ligament reconstruction for tumorous defect of the distal femur in children

**DOI:** 10.1186/s12891-022-06053-7

**Published:** 2022-12-16

**Authors:** Zhuangzhuang Li, Minxun Lu, Yuqi Zhang, Qi You, Yitian Wang, Longqing Li, Qiang Ye, Yang Wang, Yi Luo, Li Min, Yong Zhou, Chongqi Tu

**Affiliations:** 1 Department of Orthopedics, Orthopaedic Research Institute, West China Hospital, Sichuan University, No. 37 Guo Xue Xiang, Chengdu, 610041 People’s Republic of China; 2 Model Worker and Craftsman Talent Innovation Workshop of Sichuan province , West China Hospital, Sichuan University, Chengdu, People’s Republic of China

**Keywords:** Distal femur, Prosthesis, 3D-printed, Hemiarthroplasty, Limb length discrepancy

## Abstract

**Background:**

Hemiarthroplasty following tumor resection of the distal femur in children provides a chance to preserve the proximal tibial physis for limb elongation. Based on three-dimensional (3D) printing technology, the uncemented unipolar prosthesis with joint stability reinforced structures (JSRSs) was custom-designed for our cases. This study aimed to describe the design and assess the short-term outcomes of this refined prosthetic hemiarthroplasty.

**Methods:**

Seven patients (four females and three males) received 3D-printed customized uncemented unipolar prosthesis for hemiarthroplasty after removal of the distal femur, from September 2019 to October 2020 at our Orthopedics department. The limb function, growth of the preserved proximal tibial physis, joint stability, and limb length discrepancy (LLD) were assessed. Complications were recorded.

**Results:**

Six patients survived with no evidence of metastasis or local recurrence at the last follow-up, and one patient died of lung metastasis at 19 months postoperatively. Follow-up ranged from 19 to 32 months, with an average of 26 months. Elongation of the tibia was observed in all cases. At the last follow-up, four patients exhibited equal growth length compared with the healthy contralateral tibia. LLD ranged from 0.8 to 1.6 cm with a mean of 1.3 cm. The average knee range of motion was 95.3° of flexion and 4.5° of extension. All patients achieved satisfactory postoperative limb function with a mean MSTS score of 25.8. The results of the drawer, Lachman, and pivot shift tests were negative in all patients. During follow-up, painless joint space narrowing was observed in two patients. The screw for ligament fixation loosened in one of the seven patients at 17 months postoperatively. No subluxation of the joint, angular deformity, or breakage of the implant was detected in the remaining patients.

**Conclusions:**

3D-printed customized uncemented unipolar prosthesis with JSRS would be a good choice for reconstructing tumorous defect in the distal femur in children.

## Background

The distal femur is the most common location for osteosarcoma in children [1–4]. With the improvement of radiograph technology and neoadjuvant therapy, limb-salvage surgery has been the mainstream treatment [1, 5]. However, wide resection of the tumor requires removal of the distal femur to prevent recurrence and metastasis, making the sacrifice of the distal femoral physis in the affected limb inevitable. The physes of the two sides around the knee account for approximately two-thirds of the lower limb growth [2, 6]. For this reason, the preservation of the proximal tibial physis is critical to further growth of the affected limb in children who underwent wide resection of the distal femoral tumor.

The most common option for distal femoral reconstruction is the use of modular endoprosthesis because of its lower technical demand and early weight-bearing properties [7, 8], especially for adults. Nonetheless, this approach is not favorable for growing children because endoprosthetic arthroplasty would further sacrifice the physis at the unaffected proximal tibia [1, 9]. Although expandable prosthesis is designed to lengthen the limb for growing children, deep infection and soft tissue injury remain the main concern [10]. Furthermore, hemiarthroplasty with osteoarticular allograft or prosthesis is another option for reconstructing hemi-articular defect after distal femur removal in growing children. The outstanding advantage of hemiarthroplasty is the preservation of the proximal tibial physis, which attributes to 30% of the lower limb growth [11]. Osteoarticular allograft transplantation has the potential to maintain the stability of the knee joint after the surgery because of the attachment supply for ligaments reconstruction [1, 12, 13]. However, the limited donor source restricts the clinical application of this method, especially for the skeletally immature children who require high degree in allograft size and shape [9]. Even though the feasibility of prosthetic hemiarthroplasty following distal femur removal has recently been approved [2, 4], studies on this type of reconstruction are rare and the instability of the knee joint after the surgery has not been completely resolved.

In the present study, based on three-dimensional (3D) printing technology, the uncemented unipolar prosthesis with joint stability reinforced structures (JSRSs) was designed for hemiarthroplasty. Further, this study described the design and assessed the short-term outcomes of refined prosthetic hemiarthroplasty for children with osteosarcoma in the distal femur.

## Methods

### Patients

Seven patients (four females and three males) received the 3D-printed uncemented unipolar prosthesis for hemiarthroplasty following removal of the distal femur from September 2019 to October 2020 at our Department of Orthopaedics. The mean age of the patients was 9 y eras (range, 6–11 years) at the time of surgery. Patients’ demographics are shown in Table 1.

**Table 1:** Demographics and clinical data of seven distal femoral osteosarcoma patients with hemiarthroplasty

Cases	Gender/ Age at surgery (years)	Diagnosis	Length of resection (cm)	Prosthesis length (cm)	Prosthesis type	Follow-up (months)
1	F/11	IIB osteosarcoma	12.3	13.3	I	32
2	F/6	IIB osteosarcoma	14.4	15.4	I	30
3	M/9	IIB osteosarcoma	10.8	11.8	I	29
4	F/10	IIB osteosarcoma	13.4	14.4	I	27
5	M/8	IIB osteosarcoma	11.9	12.9	II	24
6	M/9	IIB osteosarcoma	11.6	12.6	II	19
7	F/11	IIB osteosarcoma	15,5	16.5	II	20

*F* Female, *M* Male, *I* first-generation hemiarthroplasty prosthesis, *II* second-generation hemiarthroplasty prosthesis

All patients underwent radiographic examinations of lesions, including X-ray examination, 3D computerized tomography (3D-CT), and magnetic resonance imaging (MRI). In addition, single-photon emission computed tomography (SPECT) or positron emission tomography/ computerized tomography (PET/CT) and biopsy were performed. All the patients were diagnosed with osteosarcoma in the distal femur, and the lesions involved the physis.

This study was approved by the Ethical Committee of our institution. Written informed consent was obtained from the patients’ parents.

### Prosthesis design and fabrication

**Fig. 1:**
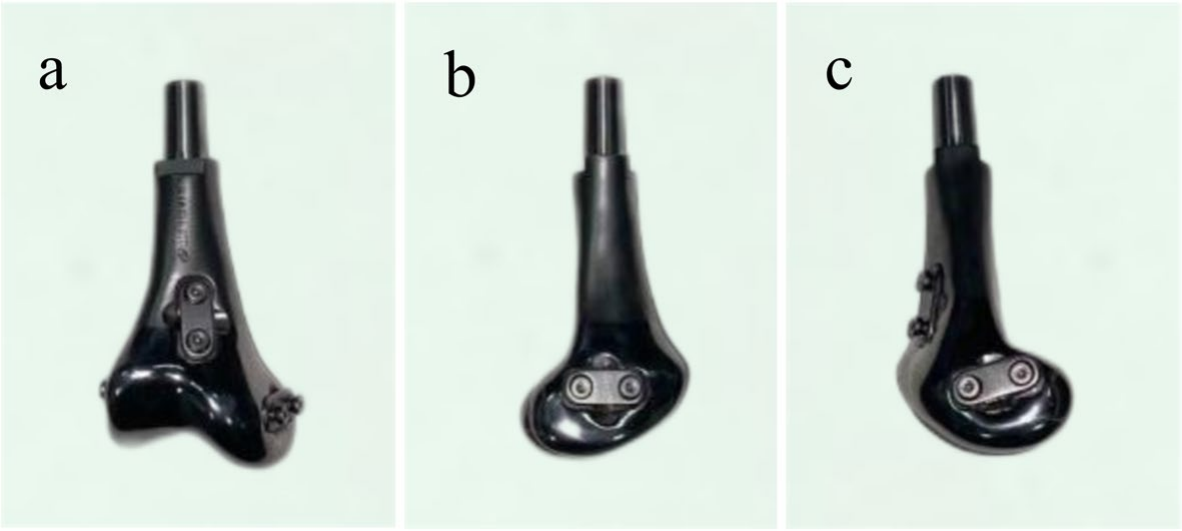
Profile of the first-generation prosthesis. **a** JSRS located on the front of the prosthetic femoral condyle was used to reconstruct anterior and posterior cruciate ligaments; (b-c) JSRSs located on medial and lateral of the prosthetic femoral condyle were used to reconstruct medial and lateral collateral ligaments. JSRS, joint stability reinforced structure

**Fig. 2:**
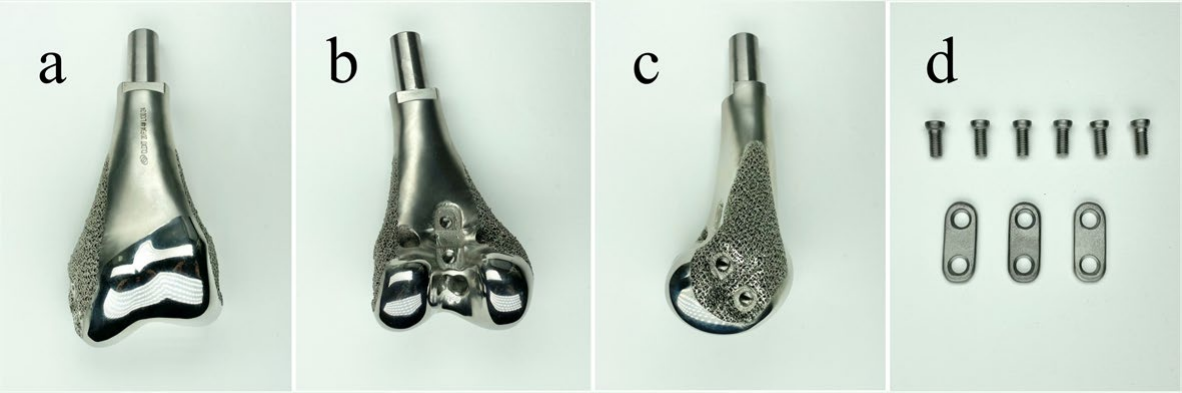
Profile of the second-generation prosthesis. **a**-**b** JSRSs were located on the posterior, medial, and lateral surfaces of the prosthetic femoral condyle; **c** Medial and lateral surfaces of the prosthetic femoral condyle have porous structures; **d** Plate screw system of JSRS. JSRS, joint stability reinforced structure

The prostheses were designed by our team and fabricated by Chunli Co., Ltd. (Tongzhou, Beijing, People’s Republic of China). Firstly, the 3D-CT data of patients was collected and imported into the Mimics V20.0 software (Materialise Corp., Belgium) to build virtual 3D tumor and bilateral femur models. The osteotomy plane was determined by the combination of MRI and CT images and set as 30 mm beyond boundary. Next, tumor resection was simulated and measured in length. The virtual 3D tumor and femur models were set as STL format and imported into Geomagic Studio software (Geomagic Inc., Morrisville, United States) for designing prosthesis. The preliminary shape of prothesis, characterized by the shape of femoral condyle, was mirrored from the contralateral femur model. After that, surface smooth of the prosthesis was performed. To improve the joint stability, JSRS, consisting of shallow groove on the prosthesis surface and cooperative plate screw system, was added on the prosthesis model. The shallow groove was designed for containing the reconstructed artificial ligament, and the plate screw system could play the role of press-fixation. The location of JSRSs were determined by referencing anatomical position of the ligaments connecting the distal femur. In detail, the medial and lateral of prosthetic femoral condyle were designed with JSRSs for reconstruction of the medial and lateral collateral ligaments. However, the position of JSRS for reconstruction cruciate ligaments varied in two generation prosthesis, locating on the anterior of prosthetic femoral condyles in the first-generation prosthesis (Fig. 1), while it was located on the posterior in the second-generation prosthesis (Fig. 2). Compared to the first-generation prosthesis, the reconstruction procedure of anterior and posterior cruciate ligaments was simplified because the artificial ligaments would not need to wrap around the prosthesis. In addition, the porous structure of the medial and lateral prosthetic femoral condyle was to facilitate the attachment of fibrous tissue (Fig. 2).

Medical-grade Ti-6Al-4 V powder was used as the printing raw material. The prosthesis was fabricated using an electron beam melting (EBM) machine (ARCAM Q10plus, Mölndal, Sweden), which is a powder bed fusion technique. The yield strength of the formed titanium alloy was approximately 1000 MPa according to the mechanical test. The prosthetic component and stem were fabricated by casting, and the stem was coated with hydroxyapatite. Considering potential growth of the contralateral femur, the prosthesis was designed 1 cm longer than the length of resection. The biomimetic femoral condyle of prosthesis could articulate with the proximal tibia. The growth of proximal tibia physis would not be affected after prosthesis implantation. Meanwhile, the repairing of collateral and cruciate ligaments could restore the stability of the knee.

### Surgical technique

**Fig. 3:**
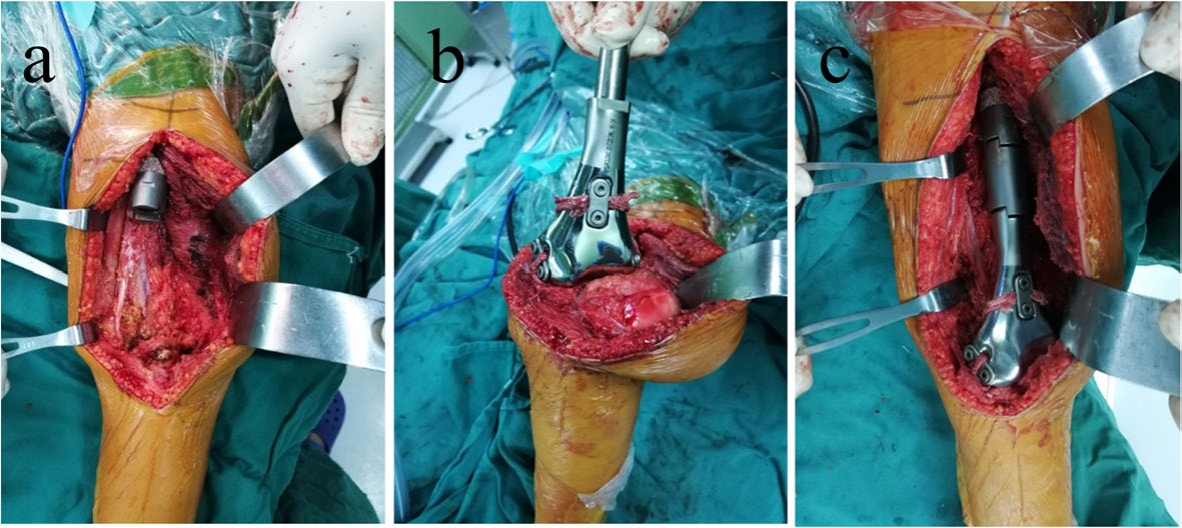
Intraoperative pictures. **a** Stem of the proximal part was press-fit inserted into the femur medulla cavity; **b** LARS ligaments were firmly anchored in shallow grooves on the surface of the prosthesis through steel plates and screws; **c** Proximal and distal parts of the prosthesis were carefully assembled. LARS, Ligament Augmentation and Reconstruction System

All surgeries were performed by the same senior doctors. The supine position and medial parapatellar arthrotomy incision were primarily used. The tumor was resected en-bloc according to the osteotomy plan as determined by preoperative imaging. The ligaments around the knee were resected at the tibia side, which remained about 1 cm in length. Subsequently, the residual femur was reamed, and the stem of the proximal part was inserted into the medullary cavity of the femur. The next step was the reconstruction of the cruciate and collateral ligaments. First, the Ligament Augmentation and Reconstruction System (LARS) ligaments were sutured to the residual ligaments. The free sides of the LARS ligaments were anchored in the shallow grooves on the surface of the prostheses through steel plates and screws. After confirming that the LARS ligaments were firmly anchored, the proximal and distal parts of the prosthesis were carefully assembled (Fig. 3).

### Postoperative management and follow-up

The affected limb was immobilized for 3 weeks postoperatively, and isometric exercises of the quadriceps were required at the same time. Walking with crutches was recommenced at the fourth week, and the weight-bearing of the affected limb increased gradually. Follow-ups for all patients were performed every 3 months in the first 2 years, every 6 months in the third year, and annually thereafter. All patients underwent physical examination and radiological assessments at each follow-up. Local recurrence and metastasis were monitored using CT. X-ray examination of the bilateral lower limbs was used to measure the limb length discrepancy (LLD) and evaluate the growth ability of the preserved tibial physis. In addition, to assess the erosion of the tibial plateau, the joint space in width based on radiology was measured. At the last follow-up, the range of motion (ROM) of the knee was measured. The postoperative function of the reconstructed limb was assessed according to the Musculoskeletal Tumor Society (MSTS) score system. The function was scored from 0 to 5 using the following six items: pain, level of activity, emotional acceptance, use of orthopedic supports, walking ability, and gait. The knee stability was evaluated by the drawer, Lachman, and pivot shift tests, and a positive result for any of these tests suggested unstable knee. The complications, such as subluxation of the joint, angular deformity, aseptic loosening, and breakage of the implants, were recorded.

## Results

**Fig. 4:**
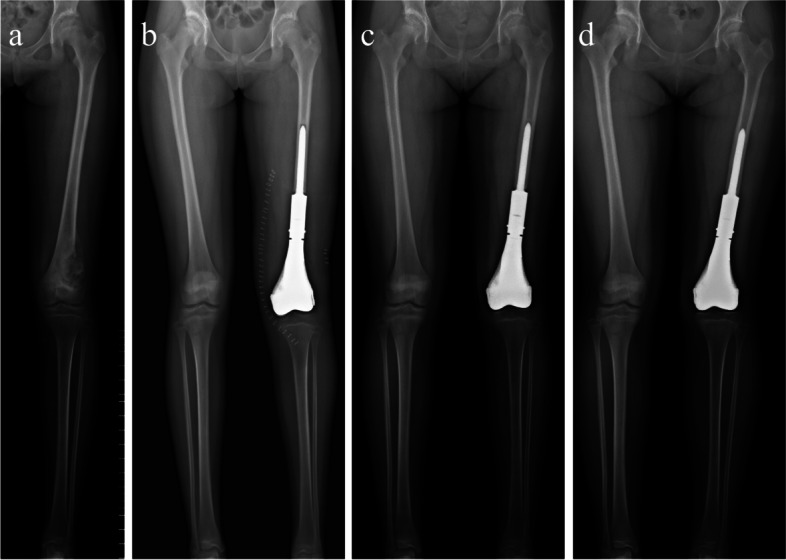
The radiograph of an eleven-year-old girl with osteosarcoma in the distal femur (case 7). **a** Preoperative radiograph showing the distal femoral physis involved in the tumor; **b** Radiograph after surgery showed that the patient underwent hemiarthroplasty with the second-generation prosthesis following tumor resection, and the prosthesis is 1 cm longer than the length of resection; **c** Eight months postoperative radiograph; **d** Radiograph 18 months after surgery showing an LLD of 0.8 cm. LLD, limb length discrepancy

**Fig. 5:**
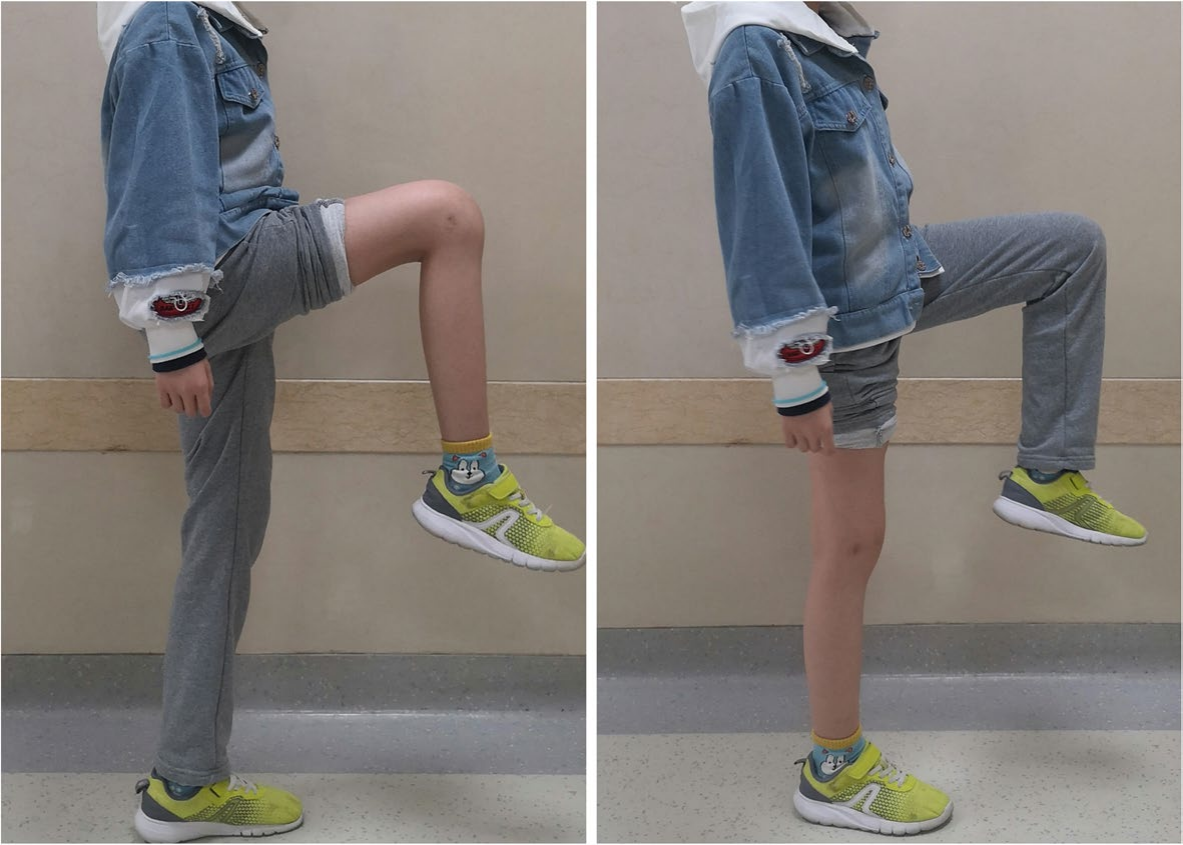
Eleven months after surgery, the knee function of one patient

**Fig. 6:**
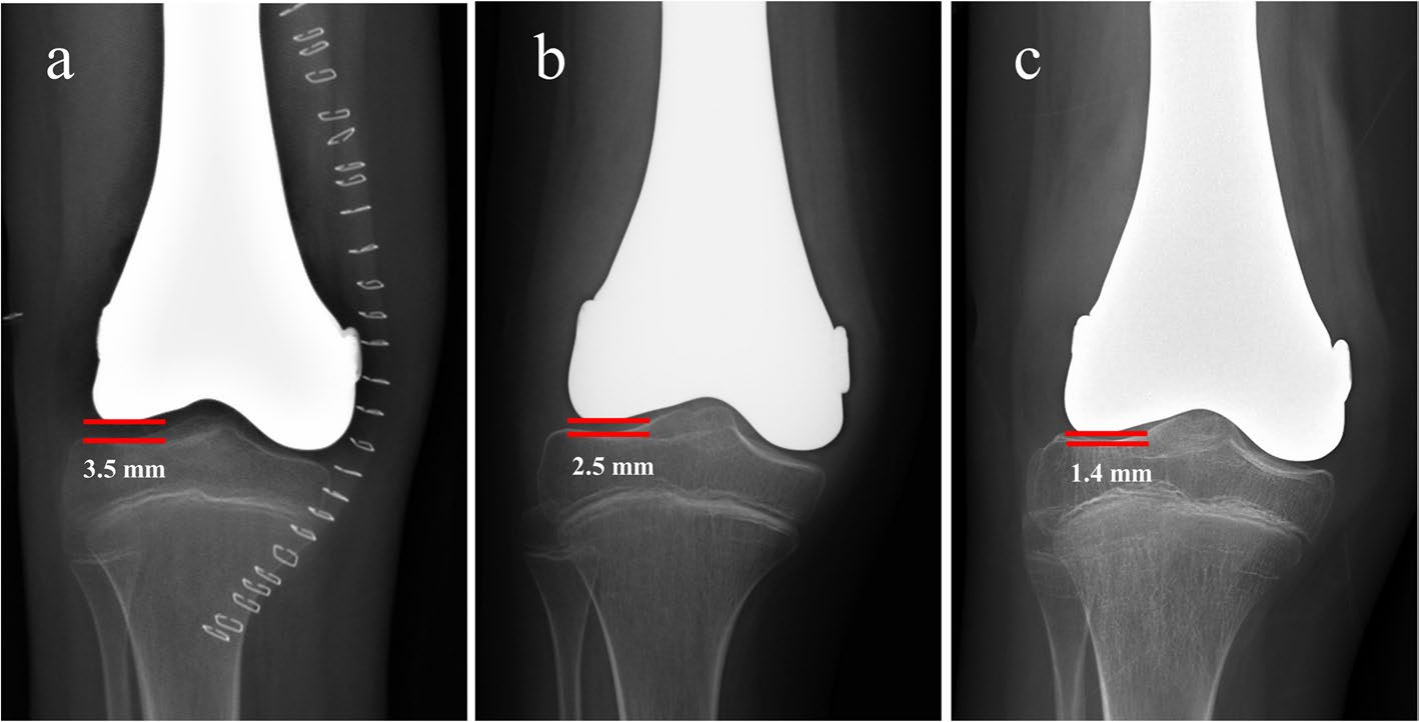
The radiograph of case 1 showing the progressing joint space narrowing. **a** After the surgery, joint space was 3.5 mm; **b** Ten months after the surgery, joint space was 2.5 mm; **c** 27 months after the surgery, joint space was 1.4 mm

Six patients survived with no evidence of metastasis or local recurrence at the last follow-up, while one patient died of lung metastasis at 19 months after the surgery. Follow-up period ranged from 19 to 32 months, with an average of 26 months. Elongation of the tibia was observed in all cases. At the last follow-up, four patients exhibited equal growth length compared with the healthy contralateral tibia (Fig. 4). The LLD ranged from 0.8 to 1.6 cm with a mean of 1.3 cm (Table 2). The average knee ROM was 95.3° of flexion and 4.5° of extension. All patients achieved satisfactory postoperative limb function with a mean MSTS score of 25.8 (Fig. 5). In addition, the results of the drawer, Lachman, and pivot shift tests were negative in all patients. During follow-up, painless joint space narrowing was observed in two patients (Fig. 6). In addition, the ligament fixation screw loosened in one of the seven patients at 17 months postoperatively. Consequently, the patient underwent reoperation to relock the loosened screw. No subluxation of the joint, angular deformity, or breakage of the implant was detected in the remaining patients.

**Table 2:** Outcomes of seven distal femoral osteosarcoma patients after hemiarthroplasty

Cases	ROM (°)		Elongation of the tibia (cm)		LLD (cm) at the last follow-up (femur/tibia)	Complications	MSTS score (final)
	Flexion	Extension	Operated	Non-operated			
1	95	5	1.9	1.9	1.4 (1.4/0)	Joint space narrowing	26
2	88	4	1.8	1.8	1.6 (1.6/0)	Joint space narrowing	25
3	89	5	1.6	1.6	1.5 (1.5/0)	None	25
4	97	5	0.8	1.0	1.1 (0.9/0.2)	None	26
5	90	3	0.9	0.9	1.1 (1.1/0)	None	26
6	-		-	-	-	Screws loosening Lung metastasis	Death
7	113	5	1.0	1.1	0.8(0.7/0.1)	None	27

*ROM* Range of motion, *MSTS* Musculoskeletal Tumor Society scoring system, *LLD* Limb length discrepancy

## Discussion

Reconstruction of the distal femoral hemi-articular defect presents huge challenges with regards to the further elongation of the affected limb, especially for skeletally immature patients. Numerous reconstruction approaches have been reported in the past; however, the optimal choice remains controversial. Expandable prosthesis is a reasonable choice to equalize limb length for growing children [1, 4]. However, the problem involved this method is the relatively high rate of complications due to frequent lengthening procedures and overstretching of the soft tissue. Zou et al. reported complications in 36% of patients who underwent expandable prosthetic replacement in their study [5]. Besides aseptic loosening and deep infection, certain specific complications were reported on using expandable prosthesis, including the patellar tendon tear, and injury of nervous peroneus communis [5]. Furthermore, a high revision rate with expandable prosthesis has been reported in previous studies; up to 48% of cases required the revision surgery [14, 15]. Hence, hemiarthroplasty with the advantage of tibial physis preservation is a reasonable option for assuring further growth of the affected limb in children. Allograft transplantation for hemiarthroplasty after tumor resection could maximize the preservation of limb growth. However, inherent limits in allograft, such as limited donor sources, potential infection transmission, and immunological rejection, are hard to solve [16]. Moreover, the relatively low survival rate of osteoarticular allograft transplantation is another concern [3]. Recently, prosthetic hemiarthroplasty, which can not only prevent allogeneic complications, but also preserve potential limb growth, has been rapidly developed for the reconstruction of the distal femur defect. Modern technology, such as computer-aided design programs and additive manufacturing, offers the possibility of designing and fabricating prostheses with complex shapes, especially for preventing a mismatch with the proximal tibia. To improve the stability of the knee joint after hemiarthroplasty, our 3D-printed uncemented unipolar prosthesis was designed with JSRS for ligament reconstruction.

In our study, satisfactory result with the mean MSTS score of 25.8 was achieved. In detail, our patients’ postoperative limb function met the requirement for the activities of daily living, such that walking and even running was possible for the patients. Chung et al. also reported the use of hemiarthroplasty after the removal of the distal femoral tumor in 12 children (aged 8–12 y) [4]. The MSTS scores of these children are comparable to those of children in our study; however, our cases involved younger age with greater levels of difficulty in the surgical procedure and postoperative management. With regard to the complications, they clarified that the instability of the knee joint after hemiarthroplasty was a serious problem, and the risk of anterior subluxation was high (up to 17%) [4]. Additionally, Yao et al. highlighted the potential risk of hemiarthroplasty impairing the joint stability in their study, with the movement and limb function limited by the multi-directional instability of the knee joint [17]. To the best of our knowledge, in previous studies on prosthetic hemiarthroplasty, the sacrifice of the ligaments around the knee was inevitable due to the loss of suture position. Consequently, the stability of the knee was impaired, and patients had to suffer from an unstable knee. Contrary to previous studies, no stability-related complications were detected in our patients after ligament reconstruction using JSRS. Furthermore, the results of the drawer, Lachman, and pivot shift tests were negative, indicating that this technique helped achieve adequate strength to maintain joint stability. In 2019, Ji et al. adopted a thin stem passing through the tibial physis to constrain the prothesis, thereby improving knee stability after hemiarthroplasty [2]. However, the growth capacity of the tibial physis was weakened in most of the patients, with only a quarter of patients having equal tibial length. In the present study, JSRS did not involve the adjacent tibia, with the LARS ligaments anchored in the shallow grooves on the surface of the prosthesis through steel plates and screws. Nevertheless, we observed painless joint space narrowing in two cases (2/7, 28.6%), and the screw for LARS ligaments fixation loosened in one case (1/7, 14.3%). Hence, joint space narrowing was the most common complication in our study, which was believed to be related to the mild wear of the tibia plateau cartilage caused by the prosthesis [18]. Regardless, the patients in this study achieved satisfactory limb function without pain, indicating that this method is worth considering for the reconstruction of distal femur defect in children.

In addition, the 3D-printed customized prosthesis helped achieve a perfect fit with the proximal tibia and was of appropriate size to cater to the small joints of children. According to previous studies, the consistency between the reconstruction implant and the joint is essential for postoperative limb function [9, 11]. However, the complex shape of the defect and the small joints of children pose challenges in achieving acceptable consistency in children using traditional prosthesis manufacturing technology. Therefore, some studies have attempted to prevent the mismatching of the implant to the knee joint by compositing the prosthesis with resurfaced allograft, in which femoral condyles were modified according to the size and shape of the defect [9, 11]; however, the composite could not satisfactorily fit the proximal tibia. Additionally, Errani et al. reported a relatively low success rate with this method, with 50% of transplants removed due to infection and allogeneic fracture [9]. In the present study, using 3D printing technology, the prosthesis was custom-designed for each patient according to the mirror model of the contralateral healthy femur. It was characterized by prosthetic femoral condyles, which helped prevent the mismatching of the prothesis to the proximal tibia. The appropriate size of prothesis for the small joints of children was also achieved with this technique.

Another reason for the satisfactory outcomes in our cases was the minimal LLD. The prosthetic hemiarthroplasty following tumor resection in the distal femur performed in this study prevented the loss of the proximal tibial physis, minimizing the potential LLD at the end of growth. In actual, the observed LLD at the last follow-up was 1 cm lower than the expected LLD, because the prosthesis was manufactured 1 cm longer than the length of the resection to compensate for potential growth of contralateral distal femoral physis. Although the prosthesis was longer than the bone defect and resulted in LLD after surgery, patients would be able to adapt to this small degree of LLD [19]. Moreover, the expected LLD and consequent damage to the postoperative limb function would consequently decrease. In the present study, the prosthesis for the distal femoral hemiarthroplasty was assembled, providing an opportunity to compensate for the LLD by replacing or adding the prosthetic component.

Our study has several limitations. First, this study was retrospective and had a small sample size. Second, our mean follow-up period was short, and the outcome of the study could possibly change with a longer follow-up duration. Finally, the present study only focused on the osteosarcoma in the distal femur, future research about different types of bone tumors with randomized controlled study need to be conducted.

## Conclusions

Hemiarthroplasty combined with ligament reconstruction led to good short-term follow-up outcomes. Therefore, 3D-printed customized uncemented unipolar prosthesis with JSRS would be a good choice for reconstructing tumorous defect of the distal femur in children.

## Data Availability

The datasets used during the current study are available from the corresponding author on reasonable request.

## Abbreviations

LLD: Limb length discrepancy
3D: Three-dimensional
CT: Computed tomography
MRI: Magnetic resonance imaging
JSRS: Joint stability reinforced structure
MSTS: Musculoskeletal Tumor Society
LARS: Ligament Augmentation and Reconstruction System
ROM: Range of motion
